# Superficial Morphea: Clinicopathological Characteristics and a Novel Therapeutic Outcome to Excimer Light Therapy

**DOI:** 10.1155/2019/1967674

**Published:** 2019-09-19

**Authors:** Al-Sadat Mosbeh, Soha Aboeldahab, Mohamed El-Khalawany

**Affiliations:** ^1^Department of Dermatology, Al-Azhar University, Cairo, Egypt; ^2^Department of Dermatology, Sohag University, Sohag, Egypt

## Abstract

**Introduction:**

Superficial morphea (SM) is an uncommon entity that was described in the literature without definitive correlation to localized scleroderma (LS) or other atrophoderma diseases.

**Aim:**

To demonstrate the clinicopathological features of SM and evaluate the efficacy of different therapeutic modalities in its management.

**Patients and methods:**

A total of 28 patients with SM were studied during the period from 2010 to 2015. Clinicopathological features and therapeutic outcomes were recorded and analyzed.

**Results:**

Clinically, SM was predominant in females (71.4%) with an average onset at 33 years of age and an average duration of 15 months. It was commonly presented as asymptomatic, darkly pigmented, and multiple and slightly indurated patches. The lesions were mostly ill-defined, large-sized, and located more on the trunk. Histologically, thickening of collagen fibers was observed either localized to the papillary dermis only (38.9%) or extended into the upper reticular dermis (61.1%). Elastic fibers were generally diminished in the upper reticular dermis while the number of fibroblasts and basal melanin pigmentation were increased in the majority of cases (92.9% and 96.4%, respectively). The most commonly associated diseases were diabetes mellitus (50%) and hepatitis C virus (HCV) infection (42.8%), and their incidence was significantly higher than that in patients with LS. Excimer light showed promising effective results in the treatment of most cases (78.9%) while the response to other modalities such as topical corticosteroid alone or in combination with tacrolimus or treatment with UVA1 alone was less effective (7.1%, 23.1%, and 5%, respectively).

**Conclusion:**

Our results proposed that SM is a distinctive clinicopathological variant and not a stage in the spectrum of LS. The novel response of SM to excimer light and not for UVA1 therapy also suggests the different therapeutic outcome of SM from LS. Although SM has a significant association with DM and HCV infection, they seem not to affect the course of the disease.

## 1. Introduction

Morphea or localized scleroderma (LS) is an uncommon connective tissue fibrosing disease that affects the skin and underlying tissue. The disease is characterized by extra deposition of collagen leading to thickening of the skin [1]. The collagen deposition usually involves the reticular dermis (classic morphea) and may extend into the subcutis (subcutaneous morphea) [2].

Superficial morphea (SM) is an uncommon entity that is characterized by localized deposition of extra collagen in the papillary and upper reticular dermis. Although there are few reports describing the clinical and pathological features of this variant, there is a debate about its relation to other atrophodermic diseases [3]. Moreover, the outcome of this disease is unclear, and there is a lack in long term follow-up studies.

In this study, we reported the clinical and histologic features of patients with SM and recorded the therapeutic response of the disease to various therapeutic modalities aiming to identify the diagnostic criteria of this entity and clarify its prognosis.

## 2. Patients and Methods

During the period from 2010 to 2015, we were able to study 28 patients diagnosed with SM. At the same period, we registered 46 patients with LS and recorded their clinicopathological features.

The diagnosis of SM was confirmed after skin biopsy and histological examination of the specimen. The histological hallmark of the disease was the presence of thickened collagen bundles in the papillary ± upper reticular dermis. Classic morphea was excluded by the absence of thickened collagen in the mid and deep reticular dermis while lichen sclerosus et atrophicus (LSA) was excluded by the absence of epidermal changes, sclerosis of the papillary dermis, and band-like lymphocytic infiltrate.

All patients on topical therapy were requested to stop the medications for two weeks prior to skin biopsy while patients on systemic therapy were excluded from the study. The study was approved by the local ethics committee and the Institutional Review Board. Informed consent was obtained from some patients for medical photography.

Clinical characteristics of the lesions including onset, course, location, morphology, and duration were recorded. Histological examination assessed the epidermal changes, morphology and density of dermal collagen, and density of fibroblast. Evaluation of morphology and density of elastic fibers was performed after special staining with Verhoeff–Van Gieson stain (VVG), orcein, and trichrome stains.

Routine laboratory investigations including complete blood count, kidney functions, liver functions, blood glucose level, erythrocyte sedimentation rate, and hepatitis B and C virus infection were performed for all patients. Other specific investigations such as antinuclear antibodies and rheumatoid factor in addition to radiological examination were performed when there was an indication for this.

The therapeutic modality was reported for each patient. As a standard treatment protocol, we started our treatment with topical application of a potent steroid for a maximum period of 6 weeks. If no response or relapse occurred, a combination therapy of tacrolimus (0.03%) and the potent steroid was used for a maximum period of 8 weeks. If no response or relapse occurred, the patient was shifted to phototherapy with UVA1 (twice per week for 6 months). Patients not responding to UVA1 were shifted to excimer light (twice per week on nonconsecutive days for 6 months).

A 308 nm xenon chloride monochromatic excimer light (MEL) (MEL@308 nm) delivery system (Excilite®, DEKA M.E.L.A, Florence, Italy) was used to irradiate the skin with an average power density of 50 mW/cm^2^ and continuous emission of 1–90 seconds. The total optical power was 2400 mW. The minimum spot size was circular of 0.8 cm^2^ while the maximum was rectangular of 30 cm^2^ (5 × 6 cm). Treatment was started with a fluence of 50 mJ/cm^2^. The average distance from the tube and the skin surface was 12–15 cm. The fluence was increased by 50 mJ/cm^2^ per session until a slight erythema appeared after 24 hours.

Follow-up of the patients was continued for one year after the end of therapy. The therapeutic response was graded as poor if there was no response, good if induration only was improved, and excellent if induration and pigmentation were improved. Patients with flare-up or relapse after good or excellent response to any therapeutic modality were shifted to the second-line treatment. The response was categorized as satisfied if good improvement was achieved without relapse or flare-up of the lesions.

## 3. Results

Out of 28 patients enrolled in this study, 20 patients were female (71.4%) with a mean age of 33 years. The mean duration of the disease was 13 months.

**Figure 1:**
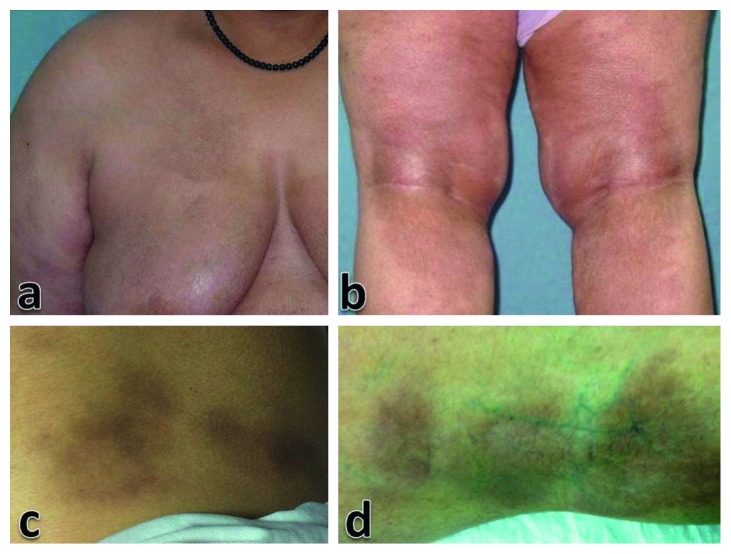
Multiple lesions of SM distributed in different locations including the trunk (a, c) and lower extremities (b, d).

**Figure 2:**
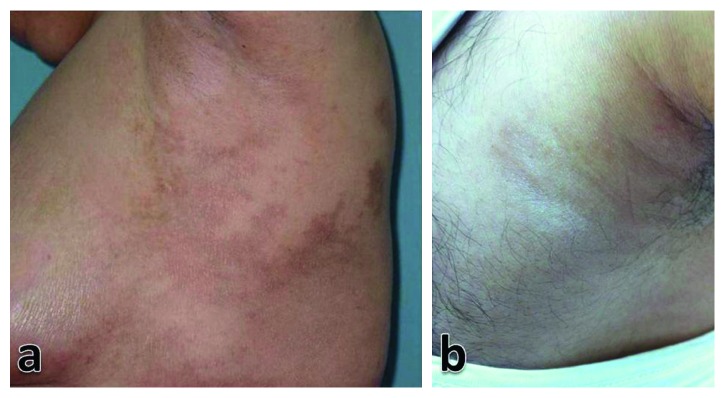
Pigmented lesions of SM characteristically involving the axillary region in female (a) and male (b) patients.

Clinical assessment of the lesions demonstrated a common presentation of asymptomatic hyperpigmented nonscaly multiple patches (Figure 1). Most of the lesions showed an ill-defined border (89.3%), mild induration (60.7%), and were located on the trunk (64.3%). Involvement of the axillary region was characteristic in 10 patients (Figure 2). There was no follicular plugging or sclerotic changes observed in any lesion.

Comparing with classic morphea, data on a total of 46 patients were collected at the same period. This disease was also common in female with a higher average age (45 years) but less duration (average 8 months). The lesions were more presented as single atrophic plaques with moderate induration (89.1%) and more distributed as disseminated lesions (Table 1).

In addition to the early stage of classic morphea, the other differential diagnoses of SM at the time of presentation were hyperpigmented mycosis fungoides, lichen planus pigmentosus, and postinflammatory hyperpigmentation.

**Figure 3:**
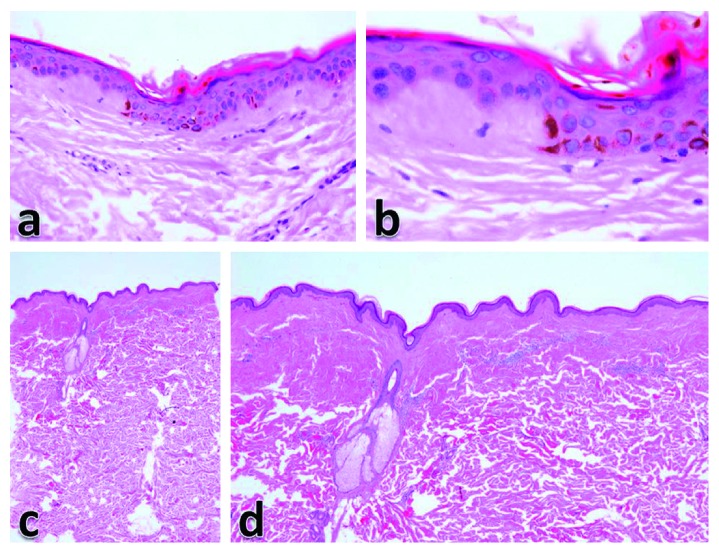
Histologic assessment of SM may show thickened collagen observed only in the papillary dermis ((a) H&E ×100 and (b) H&E ×200) or thickened collagen observed in both papillary and upper reticular dermis ((c) H&E ×20 and (d) H&E 100).

**Figure 4:**
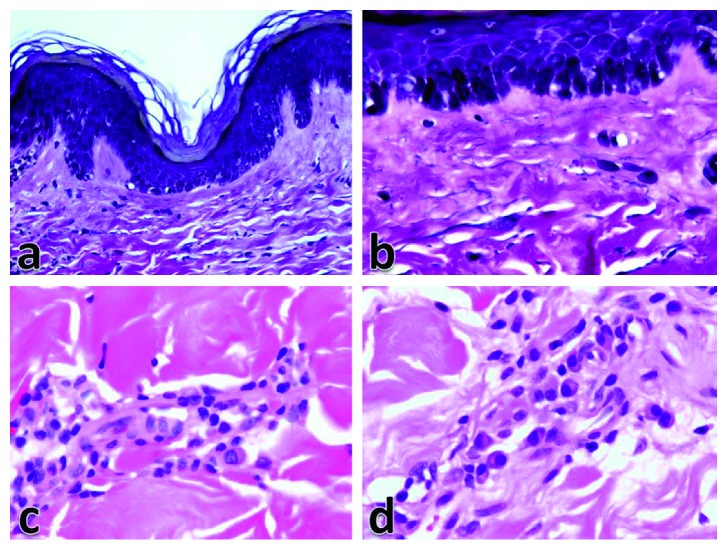
Elastic fibers are diminished in the upper dermis ((a) VVG ×400) and usually arranged as transverse fine short streaks ((b) VVG ×1000). The inflammatory infiltrate is usually formed of perivascular lymphocytes with few plasma cells ((c) H&E ×1000), but predominance of plasma cells was also seen ((d) H&E ×1000).

Histologic assessment of the lesions showed normal epidermal thickness or mild epidermal thinning. Increased basal melanin pigmentation was a predominant finding but mostly in a localized fashion. The thickening of collagen fibers was observed in two patterns (Figure 3), either localized to the papillary dermis only (38.9%) or observed in both papillary and upper reticular dermis (61.1%). Elastic fibers were generally diminished in the upper reticular dermis compared with the deep reticular dermis. The elastic fibers were observed as fine transverse short streaks parallel to the epidermis (Figures 4(a) and 4(b)). The dermal fibroblasts were mildly increased in number in most patients (71.4%). The inflammatory infiltrate is usually mild to moderate and concentrated mainly around the middermal blood vessels. Although the main inflammatory cells were lymphocytes, some cases showed predominance of plasma cells (Figures 4(c) and 4(d)).

The most associated diseases with SM were diabetes mellitus (DM) (50%), HCV infection (42.8%), and hypertension (21.4%). In comparison with classic morphea, HCV infection was less commonly associated while connective tissue diseases were more commonly associated with classic morphea. There was no significant difference between both diseases in relation to diabetes mellitus, hypertension, and chronic renal diseases as shown in Table 1.

The therapeutic response was satisfied with topical steroid therapy in 7.1%, with topical combination therapy (steroid and tacrolimus) in 23.1%, with UVA1 phototherapy in 5%, and with excimer light therapy in 78.9% (Table 2).

## 4. Discussion

Superficial morphea is a unique form of morphea which was described initially by McNiff et al. [4]. Most of the previous reports described superficial morphea as pigmented patches or mildly indurated plaques. The lesions are usually multiple and distributed mainly on the trunk and intertriginous areas [5]. However, atypical presentation was also described as reported by Vučićević Boras et al. who described a case of SM in the lips and gingiva [6].

Although SM in previous reports was restricted as a disease of women, Srinivasan and DiMaio described a novel case in a male patient [7]. Our results were concomitant with previous reports, and there was agreement of the predominance of the disease among females. In spite of the predominance of SM in young age group of our patients, the disease was described in both old and young-aged patients [5, 8].

In this study, we reported an important association of SM with other diseases, more significantly DM and HCV infection. It was previously reported that morphea may coexist with other autoimmune diseases such as DM [9], and there is a close association between morphea and chronic HCV infection [10]. It was proposed that hepatitis virus may play a significant role in altering the immune system and predispose for development of other immunological skin diseases such as morphea [11].

The commonest clinical presentation in this study was multiple hyperpigmented mildly indurated large-sized patches which were distributed mainly on the trunk. Classically, SM is characterized by hypopigmented to hyperpigmented lesions located predominantly in a symmetric fashion at intertriginous sites [5]. A hint of violaceous erythema and occasional minimally atrophic, hypopigmented discrete areas within the patches may be observed [7].

The coexistence of SM with other skin diseases is rare and only reported by Saleh et al. in a patient with psoriasis vulgaris [12]. Although we did not report any chronic skin disease associated with SM, McNiff et al. found lesions of classic morphea in 50% of patients with SM [4]. Unlike SM, classic morphea is frequently reported in association with other autoimmune skin diseases such as vitiligo, LSA, and discoid lupus erythematosus [13–15], the findings that were also recorded in our patients.

Although classic morphea differs clinically and histologically from SM, the relationship between both diseases was not clarified. SM is either a strictly unique variant of morphea or a phase in the progression of classic morphea. Lack of long-term follow-up of SM in most of the previous reports postponed the exact correlation of both entities. It was proposed that SM considered an abortive form of scleroderma in which sclerosis failed to develop [3].

There was also a doubt about the relation of SM to atrophoderma Pasini-Pierini (APP). Some authors believed that both entities are similar or identical, especially the atrophic stage following the regression of morphea. This concept was proposed because both entities are characterized by a chronic benign course with favourable outcome and usually do not require treatment and do not produce disability [3].

However, other authors believed that SM is not identical to APP because it usually affects an older age group, and the lesions lose the clinical depression “cliff sign” which is a characteristic of atrophoderma. Moreover, SM differs histologically from APP by the abnormality of elastic fibers, sclerosis of collagen, and presence of active inflammation in the regressed lesion [8].

Although lichen sclerosus et atrophicus (LSA) is one of the important clinical differential diagnoses of SM, the histologic characteristics of LSA in the form of epidermal thinning, follicular hyperkeratosis, vacuolar alteration of the basal layer, subepidermal edema, and striking loss of elastic fibers easily differ between both diseases [16].

Although Walker et al. in a recent report reviewing the clinicopathological criteria of 83 patients with morphea found that histological finding of top-heavy sclerosis pattern (involvement of the papillary dermis and the superficial reticular dermis) was significantly associated with patients who had clinical features of lichen sclerosus accompanying morphea, this pattern was also more encountered in other clinical forms of morphea, especially generalized and linear types but without significant correlation [17].

They also reported that top-heavy patterns of sclerosis is predominant in patients with isomorphic morphea which is located in sites of chronic friction such as the waistband area, the observation that was also reported in about 1/3 of our patients in the axillary region and reported also by Srinivasan and DiMaio but in the crural area [7].

Patients with SM usually complain of a cosmetic problem of the lesions rather than other symptoms that may occur in classic morphea such as pain, tightness, and functional impairment or disability. This was explained by the correlation of these symptoms to the deep pattern of sclerosis that involve the deep reticular dermis and subcutis which is giving a higher incidence of perineural inflammation and sclerosis, resulting in different grades of signs and symptoms [18]. Thus, Walker et al. proposed that biopsy in morphea is not only indicated for diagnosis but also for assessment of the disease by determining the severity of inflammation and level of sclerosis that will determine the therapeutic strategy with consideration of the clinical assessment [17].

The treatment of SM is somewhat controversial, and until now there was no standard treatment for this disease. It was proposed that treatments that have been used successfully in classic morphea can be implemented in SM [5]. In this study, we found a novel good response to excimer light. Although UVA1 showed good improvement in three patients, relapse or flare-up of the lesions was observed in two patients.

Topical calcipotriene (0.005%) was reported as an effective treatment of SM localized to the crural area within 3 months of treatment [7]. In another case, topical tacrolimus (0.1%) showed mild improvement in a female patient with multiple lesions on the trunk while narrow-band UVB and clobetasol (0.05%) were ineffective [5].

These results, in addition to ours, suggested that SM is more likely to be a unique clinicopathological variant of morphea and not a separate entity or correlated to other atrophodermic diseases.

## 5. Conclusion

To the best of our knowledge, this is the first study in the literature describing the clinicopathologic and therapeutic outcomes of SM in a large number of patients. Our results proposed that SM is a distinctive clinicopathological variant and not a stage in the spectrum of LS. The novel response of SM to excimer light and not for UVA1 therapy also suggests the different therapeutic outcome of SM from LS. Although SM has a significant association with DM and HCV infection, they do not seem to affect the course of the disease.

## Figures and Tables

**Table 1:** Comparison between demographic data and clinical features in superficial morphea and localized scleroderma.

Demographic and clinical data	Superficial morphea (*n* = 28)	Localized scleroderma (*n* = 46)
*Age*		
Range	26–47	19–61
Mean ± SD	33 ± 2.11	45 ± 8.7

*Sex*		
Male	**8** (28.6%)	**19** (41.3%)
Female	**20** (71.4%)	**27** (58.7%)
M/F ratio	1 : 2.5	1 : 1.4

*Duration of the disease (m)*		
Range	7–22	4–13
Mean ± SD	15 ± 3.56	8 ± 3.82

*Clinical features of the lesion (s)*		
Number (single/multiple)	**7** (25%)/**21** (75%)	**24** (52.2%)/**22** (47.8%)
Border (well defined/ill-defined)	**3** (10.7%)/**25** (89.3%)	**10** (21.7%)/**36** (78.3%)
Size (small <5 cm/large >5 cm)	**5** (17.9%)/**23** (82.1%)	**11** (23.9%)/**35** (76.1%)
Induration (no/mild/moderate)	**11** (39.3%)/**17** (60.7%)/**0**	**0**/**5** (10.9%)/**41** (89.1%)
Location (trunk/upper limb/lower limb/disseminated)	**18** (64.3%)/**2** (7.1%)/**7** (25%)/**1**(3.6%)	**11** (23.9%)/**13** (28.3%)/**15** (32.6%)/**19** (41.3%)

*Associated diseases*		
Diabetes mellitus	**14** (50%)	**20** (43.5%)
Hypertension	**6** (21.4%)	**8** (17.4%)
Hepatitis C virus infection	**12** (42.8%)	**10** (21.7%)
Chronic renal disease	**4** (14.3%)	**6** (13%)
Other connective tissue disease	**1** (3.6%)	**7** (15.2%)
Total (positive association)	**24 (85.7%)**	**37 (80.4%)**

**Table 2:** Therapeutic modalities and follow-up of 28 patients with superficial morphea.

Treatment modality	Topical therapy (potent steroid) (*n* = 28)	Topical combination therapy (steroid + tacrolimus) (*n* = 26)	Phototherapy (UVA1) (*n* = 20)	Phototherapy (excimer light) (*n* = 19)
*Duration*				
Range (w)	4–6	4–8	12–24	12–24
Mean ± SD	5 ± 0.21	6 ± 0.73	18 ± 1.56	17 ± 2.8

*Response*				
Poor	**20** (71.4%)	**15** (57.7%)	**17** (85%)	**3** (15.8%)
Good	8	10	3	14
Excellent	0	1	0	2

*Flare-up/relapse*				
Number	**6**/8	**5**/11	**2**/3	1/16
Duration (m)	4–7	1–4	—	—

Final satisfied response	2/28 **(7.1%)**	6/26 **(23.1%)**	1/20 **(5%)**	15/19 **(78.9%)**

## Data Availability

The data used to support the findings of this study are included within the article.
